# Prevalence of Waterpipe Tobacco Smoking Among Population Aged 15 Years or Older, Vietnam, 2010

**DOI:** 10.5888/pcd10.120100

**Published:** 2013-04-18

**Authors:** Le Thi Thanh Xuan, Hoang Van Minh, Kim Bao Giang, Pham Thi Quynh Nga, Phan Thi Hai, Nguyen Thac Minh, Jason Hsia

**Affiliations:** Author Affiliations: Hoang Van Minh, Kim Bao Giang, Institute of Preventive Medicine and Public Health, Hanoi Medical University, Hanoi, Vietnam; Pham Quynh Nga, World Health Organization Office in Vietnam, Hanoi, Vietnam; Phan Thi Hai, Nguyen Thac Minh, Vietnam Steering Committee on Smoking and Health, Hanoi, Vietnam; Jason Hsia, Centers for Disease Control and Prevention, Atlanta, Georgia.

## Abstract

**Introduction:**

The prevalence of waterpipe tobacco smoking is increasing globally and is associated with adverse outcomes requiring tobacco control interventions. We estimated the prevalence of waterpipe tobacco use among adult populations in Vietnam in 2010 and examined its association with sociodemographic factors.

**Methods:**

We used data from the Global Adult Tobacco Survey (GATS) conducted in Vietnam in 2010. GATS surveyed a national representative sample of adults aged 15 years or older from 11,142 households by using a 2-phase sampling design analogous to a 3-stage stratified cluster sampling. Descriptive statistical analyses and multivariate logistic regression modeling were conducted.

**Results:**

A total of 6.4% of Vietnamese aged 15 years or older (representing about 4.1 million adult waterpipe smokers) reported current waterpipe tobacco smoking. The prevalence of waterpipe tobacco smoking was significantly higher among men than women (13% vs 0.1%). Area of residence (rural or urban), age group, asset-based wealth quintile, and geographic region of residence were significantly associated with waterpipe tobacco smoking among men. The significant correlates of current waterpipe tobacco smoking among men were lower education levels, being middle-aged (45–54 years), lower asset-based wealth levels, living in rural areas, not living in the South East and the Mekong River Delta geographic regions, and the belief that smoking does not causes diseases.

**Conclusion:**

Rural dwellers who are poor should be targeted in tobacco control programs. Further studies are needed that examine perceptions of the adverse health effects and the cultural factors of waterpipe tobacco smoking.

## Introduction

A tobacco waterpipe (also known as hookah, narghile, and shisha) is a device that allows tobacco smoke to pass through water before inhalation (1). Waterpipe smoke contains harmful constituents, and evidence links waterpipe smoking to various life-threatening conditions, including pulmonary disease, coronary heart disease, pregnancy-related complications (2), cancers, oral dysplasia, and infertility (3). Despite these negative health effects, the prevalence of waterpipe tobacco smoking is increasing globally, particularly among youth (4–6). Previous studies found that because of the belief that waterpipe tobacco is less harmful than other forms of tobacco, such as cigarettes (7–9), waterpipe smoking is showing signs of a global epidemic with serious implications for public health and tobacco control worldwide (1–5). Waterpipe use has been declared a public health problem by the World Health Organization and other authorities (2–5,8,10). Therefore, estimates of the prevalence of waterpipe tobacco smoking among populations represent a key indicator to monitor in tobacco control programs nationally and internationally, and to evaluate the achievement of tobacco control policies.

Vietnam is among countries with the highest smoking rates in the world. The prevalence of tobacco smoking in 2001–2002 was estimated at 51.2% (11). Like elsewhere in the world (10,12), in Vietnam waterpipe smoking follows cigarette smoking as the most common method of tobacco use (13). According to a study among male smokers in Vietnam during 2001–2002, 69.1% smoked cigarettes only, 23.2% smoked waterpipe only, and 7.7% reported using both products (11).

Tobacco control has received recent attention in Vietnam. The Vietnamese government’s readiness to curb the tobacco use epidemic was well reflected in 2 Vietnamese legislative actions (14,15). Vietnam signed the Framework Convention on Tobacco Control on August 8, 2003, and ratified it on November 17, 2004 (16).

Although the number of research projects on tobacco use in Vietnam has been increasing rapidly, there remains a lack of updated and national representative reports on the pattern of waterpipe tobacco smoking and its correlates. We estimated the prevalence of waterpipe tobacco smoking among adult populations in Vietnam and examined its associated socioeconomic factors. These findings provide a scientific basis for establishing effective interventions and creating policies specific to Vietnam for reducing waterpipe smoking.

## Methods

Data used in this article were obtained from the Global Adult Tobacco Survey (GATS) conducted in Vietnam in 2010. GATS is a household survey that was launched in February 2007 as a new component of the ongoing Global Tobacco Surveillance System (GTSS) (17). The standard GATS questionnaire was designed to collect data on adult tobacco use and key tobacco control measures. GATS in Vietnam was designed to be a nationally representative survey of all noninstitutionalized men and women aged 15 years or older who considered Vietnam to be their primary place of residence and excluded people living in hospitals, military barracks, and prisons. The GATS questionnaire has been adapted to the Vietnamese context, and the final version of the questionnaire was approved by the GATS Questionnaire Review Committee (17).

A total of 11,142 households were selected for this survey by using a 2-phase sampling design analogous to a 3-stage stratified cluster sampling. Strata used were districts and areas (urban or rural). The phase I sample was the 15% master sample of enumeration areas that the General Statistics Office, Vietnam, created by using the 2009 census. The phase II sample was a probability sample of enumeration areas from the 15% master sample. 

Data collection was conducted by the General Statistics Office (GSO) under the co-supervision of the World Health Organization in Vietnam, the Vietnam Steering Committee on Smoking and Health (VINACOSH), and Hanoi Medical University. Institutional review board approval was from the Ministry of Health. Twenty-six data collection teams were involved in GATS Vietnam 2010. Each team consisted of 1 team leader and 4 interviewers to ensure close supervision and collection of high-quality data. Team members had computer skills and previous experience in conducting GSO household-based surveys, especially GSO health-related surveys. In addition to the qualifications required of interviewers, GATS team leaders were experienced in using computers and handheld computer devices and had previous experience working with local authorities.

Each interviewer and team leader had 1 HP iPAQ 210 (Hewlett Packard, Palo Alto, California), which they used for capturing data. A case file containing addresses and names of the households assigned to the interviewer was preloaded in the iPAQ before field work began. The iPAQ randomly selected 1 respondent in each household for individual interview. All responses were entered in the iPAQ by using a stylus for touching the keypad on the screen. Data collection was conducted from March 22 through May 13, 2010, in all 63 provinces of Vietnam.

Among the 11,142 sampled households, 10,383 provided complete information, giving a household response rate of 93.2%. Among 10,383 people selected from the completely screened households, 9,925 provided complete information, for an individual-level response rate of 95.6%, representing an estimated 64.3 million adults aged 15 years or older. Of the study population, 48.6% were men and 51.4% were women.

In our study, the dependent variable was current waterpipe smoking. The questionnaire asked 1) Do you currently smoke tobacco daily, occasionally, or not at all? and 2) On average in 1 day or in 1 week, in how many waterpipe sessions do you smoke tobacco? Respondents who said that they currently smoke tobacco daily or occasionally to question 1 and said that they smoke at least 1 waterpipe session per day or per week to question 2 were classified as current waterpipe smokers. The nonwaterpipe tobacco smokers were those who reported that they did not smoke waterpipe tobacco at present but they may or may not smoke other tobacco products. The independent variables were sex, age group, asset-based wealth index quintile (this index was constructed by using household assets, utilities, and housing construction as variables in principal-components analysis to compute a wealth index for each household), ethnicity, occupation, marital status, education level, urban or rural area of residence, geographic region of residence, knowledge of health harms or diseases caused by smoking, and access to information (health warnings or advertisement). The question used to determine knowledge of health harms or diseases was “According to you, does smoking tobacco (cigarette, pipe tobacco, pipe, terracotta bowl pipe) cause dangerous diseases?”

Both descriptive and analytical analyses were carried out using Stata10 software (Stata Corp LP, College Station, Texas). We used descriptive analyses to determine the prevalence of current waterpipe tobacco smoking among populations. On the basis of our hypothesis on the relationships between waterpipe tobacco smoking and demographic variables, we performed multivariate logistic regression modeling (presented as odds ratio [OR] and corresponding 95% confidence interval [CI]) to examine the association between current waterpipe tobacco use among men with selected sociodemographic factors. Because education level was reported only among respondents aged 25 years or older, we constructed 2 models: 1) in model A, for all men aged 15 years or older, education was excluded; and 2) in model B, for men aged 25 years or older, education was included as an independent variable. The sampling design was fully taken into consideration in data analysis. Weights were used in all computations. We used χ^2^ tests to test significant difference among groups. A significance level of *P* < .05 was used.

## Results

In 2010, a total of 6.4% of adults in Vietnam aged 15 years or older reported current waterpipe tobacco use (representing about 4.1 million adult waterpipe smokers) (Table 1). The percentage that exclusively smoked waterpipe tobacco was 3.8% versus 2.6% for mixed waterpipe and other tobacco use. Among current smokers who did not smoke waterpipe tobacco, 18.5% reported using other tobacco products.

**Table 1:** Prevalence of Waterpipe Tobacco (WT) Smoking Among Adults Aged 15 Years or Older, Global Adult Tobacco Survey, Vietnam, 2010

WT Smoking Status	All WT Smokers, % (95% CI)	Exclusively WT Smokers, % (95% CI)	WT and Other Tobacco Smokers,^a^ % (95% CI)
Current WT smoker^b^	6.4 (5.9–6.9)	3.8 (3.4–4.2)	2.6 (2.3–2.9)
Daily WT smoker	5.4 (5.0**–**5.8)	3.3 (3.0**–**3.7)	2.1 (1.8**–**2.3)
Occasional WT smoker	1.0 (0.8**–**1.2)	0.5 (0.3**–**0.6)	0.5 (0.4**–**0.7)
Occasional WT smoker, formerly daily	0.3 (0.2**–**0.4)	0.1 (0.0**–**0.1)	0.2 (0.1**–**0.3)
Occasional WT smoker, never daily	0.7 (0.6**–**0.9)	0.4 (0.2**–**0.5)	0.4 (0.3**–**0.5)

Abbreviation: CI, confidence interval.

a Individuals currently used waterpipe in addition to other tobacco products.

b Current WT use includes both daily and occasional (less than daily) use.

The prevalence of waterpipe tobacco smokers among men was significantly higher than among women (13.0% vs 0.1%) (Table 2). By age, the prevalence of waterpipe tobacco smoking did not show any specific pattern. The prevalence in the group aged 45 to 54 years was the highest (10.1%), and the group aged 15 to 24 years was the lowest (3.0%). The more highly educated group (college or above) had the lowest prevalence of waterpipe tobacco smokers (2.7%) and the group with lower secondary education had the highest (11.0%) (Table 2). By occupation, the prevalence of current waterpipe tobacco smoking was highest among people with unstable jobs (8.8%) and lowest among students (0.7%). The prevalence of waterpipe tobacco smoking was lowest among the richest group (2.0%) and highest among the poorest group (8.8%). By ethnicity, the prevalence among ethnic minorities was significantly higher than that of Kinh (10.8% vs 5.6%), who make up the majority (approximately 85%) of the population of Vietnam.

**Table 2:** Smoking Status of Waterpipe Tobacco (WT) Smoking Among Adults Aged 15 Years or Older, by Sociodemographic Characteristics, Global Adult Tobacco Survey, Vietnam, 2010^a^

Variable	All WT Smokers, % (95% CI)	Exclusively WT Smokers, % (95% CI)	WT and Other Tobacco Smokers, % (95% CI)
**Sex**			
Male	13.0 (12.0–14.0)	7.7 (6.9–8.4)	5.4 (4.7–6.0)
Female	0.1 (0.0–0.2)	0.1 (0.0–0.2)	0.0 (0.0–0.0)
**Age, y**			
15–24	3.0 (2.1–3.8)	1.4 (0.8–1.9)	1.6 (1.0–2.2)
25–34	6.1 (5.1–7.1)	2.8 (2.1–3.5)	3.3 (2.5–4.1)
35–44	8.2 (7.0–9.3)	4.3 (3.5–5.2)	3.8 (3.0–4.6)
45–54	10.1 (8.8–11.5)	7.6 (6.4–8.9)	2.5 (1.8–3.2)
55–64	8.1 (6.5–9.8)	5.1 (3.8–6.5)	3.0 (2.0–4.1)
≥65	4.7 (3.5–6.0)	3.9 (2.7–5.0)	0.8 (0.3–1.4)
**Education^b^**			
Primary or less	6.8 (6.0–7.6)	4.3 (3.7–4.9)	2.5 (2.0–3.0)
Lower secondary	11.0 (9.6–12.3)	6.8 (5.7–8.0)	4.1 (3.2–5.0)
Upper secondary	6.7 (5.1–8.2)	3.2 (2.1–4.3)	3.5 (2.3–4.6)
Some college or more	2.7 (1.8–3.6)	1.5 (0.8–2.2)	1.2 (0.6–1.8)
**Occupation^c^**			
Government staff	3.8 (2.6–4.9)	1.6 (0.8–2.4)	2.1 (1.2–3.0)
Nongovernment staff	1.9 (0.8–2.9)	0.9 (0.2–1.6)	1.0 (0.2–1.7)
Unstable job	8.8 (8.1–9.6)	5.2 (4.6–5.8)	3.7 (3.2–4.1)
Student	0.7 (0.1–1.3)	0.1 (0.1–0.3)	0.6 (0.1–1.2)
Other	3.7 (2.8–4.5)	2.9 (2.1–3.7)	0.8 (0.4–1.2)
**Wealth quintile^d^**			
1st (lowest)	8.8 (7.6–10.1)	5.8 (4.8–6.8)	3.1 (2.4–3.8)
2nd	8.8 (7.5–10.1)	5.2 (4.1–6.2)	3.6 (2.7–4.5)
3rd	6.8 (5.5–8.0)	4.3 (3.3–5.3)	2.5 (1.7–3.2)
4th	5.0 (4.1–5.9)	2.4 (1.8–3.1)	2.6 (1.9–3.3)
5th (highest)	2.0 (1.4–2.6)	1.0 (0.6–1.4)	1.1 (0.6–1.5)
**Ethnicity^e^**			
Kinh	5.6 (5.1–6.1)	3.3 (2.9–3.7)	2.3 (2.0–2.6)
Other	10.8 (9.2–12.4)	6.5 (5.2–7.8)	4.3 (3.3–5.4)
**Marital status**			
Single	3.0 (2.2**–**3.8)	1.1 (0.6–1.6)	1.9 (1.3–2.6)
Married	8.1 (7.4**–**8.7)	5.0 (4.5–5.5)	3.1 (2.7–3.5)
Separated	4.7 (0.0**–**9.9)	0.0 (0.0–0.0)	4.7 (0.0–9.9)
Divorced	2.2 (0.0**–**4.6)	1.8 (0.3–4.0)	0.4 (0.0–1.3)
Widowed	2.4 (1.3**–**3.5)	2.0 (1.0–2.9)	0.4 (0.0–0.9)
**Area of residence**			
Urban	2.5 (2.1**–**2.9)	1.2 (0.9–1.5)	1.3 (1.0–1.7)
Rural	8.1 (7.4**–**8.9)	5.0 (4.3–5.6)	3.2 (2.7–3.7)
**Geographic region of residence**			
Red River Delta	11.8 (10.5–13.1)	6.5 (5.5–7.6)	5.2 (4.3–6.1)
Northern midland and mountain	15.2 (13.3–17.0)	8.7 (7.3–10.2)	6.5 (5.2–7.7)
North central and central coastal	5.3 (4.4–6.2)	4.0 (3.2–4.8)	1.3 (0.9–1.8)
Central highlands	3.9 (2.5–5.3)	2.3 (1.2–3.3)	1.7 (0.7–2.6)
South East	0.7 (0.3–1.1)	0.2 (0–0.3)	0.6 (0.2–0.9)
Mekong River Delta^f^	0	0	0

Abbreviation: CI, confidence interval.

a All *P* values were <.001, χ^2^ test.

b The highest educational level that the respondent completed: primary or less is equivalent to less than 6th grade; lower secondary is equivalent to finishing 6th to 9th grade; upper secondary is equivalent to finishing 10th to 12th grade; some college and above is study after upper secondary graduation.

c Occupation based on respondent’s reported main work status over the past 12 months. Unstable jobs were defined as freelance worker and self-employed. This is created based on asset wealth index.

d This index was constructed by using household assets, utilities, and housing construction as variables in principal-components analysis and by computing a wealth index for each household surveyed.

e Based on the ethnic group that the respondent reported belonging to. Approximately 85% of the population of the country is Kinh.

f No one in the sample population in the Mekong River Delta region reported WT smoking.

Married people smoked waterpipe tobacco at the highest level (8.1%), and divorced people at the lowest level (2.2%). By area of residence, a higher proportion of rural dwellers smoked waterpipe than those in urban areas (8.1% vs 2.5%). In terms of geographic region, people living in the Northern midland and mountain region had the highest prevalence of waterpipe smoking (15.2%). None of the study population from the Mekong River Delta reported current waterpipe smoking.

Logistic regression analysis of data for men showed that current waterpipe tobacco use was not associated with ethnicity, marital status, occupation, belief that smoking harms health or belief that smoking causes disease, access to tobacco health warnings or advertisements, or education. In both models, we found a significant association between waterpipe tobacco smoking and age group, wealth quintile, area of residence, geographic region of residence, and the belief that smoking causes disease (Table 3).

**Table 3:** Logistic Regression Analysis of the Association Between Current Waterpipe Tobacco Smokers With Selected Sociodemographic Factors, Among Men Aged 15 Years or Older, Global Adult Tobacco Survey, Vietnam, 2010

Variable	Model A (Education Excluded, Men Aged ≥15 Years)		Model B (Education Included, Men Aged ≥25 Years)	

	OR (95% CI)	*P* Value^a^	OR (95% CI)	*P* Value^a^
**Age, y**				
15–24	Reference		NA	
25–34	1.4 (0.8–2.7)	.24	Reference	
35–44	1.8 (0.9–3.4)	.08	1.2 (0.8–1.8)	.28
45–54	2.6 (1.3–4.9)	.005	1.8 (1.2–2.6)	.003
55–64	1.8 (0.9–3.7)	.12	1.3 (0.8–2.0)	.30
≥65	0.8 (0.4–1.8)	.68	0.6 (0.3–1.0)	.05
**Occupation^b^**				
Government staff	Reference		Reference	
Nongovernment staff	0.6 (0.2–1.6)	.33	0.5 (0.2–1.3)	.15
Unstable job	1.8 (1.0–3.5)	.06	1.4 (0.7–3.1)	.37
Student	0.3 (0.1–1.0)	.05	0^c^	
Other	1.5 (0.7–3.1)	.33	1.2 (0.5–2.7)	.74
**Wealth quintile^ad^**				
1st (lowest)	3.8 (2.2–6.4)	<.001	3.2 (1.8–5.7)	<.001
2nd	3.2 (1.9–5.3)	<.001	2.9 (1.7–5.1)	<.001
3rd	2.7 (1.6–4.5)	.005	2.1 (1.2–3.6)	.04
4th	1.6 (1.0–2.7)	.34	1.4 (0.8–2.4)	.64
5th (highest)	Reference		Reference	
**Ethnicity^e^**				
Kinh	Reference		Reference	
Other	1.0 (0.7–1.5)	.89	1.0 (0.7–1.5)	.98
**Marital status**				
Single	0.7 (0.4–1.1)	.14	0.8 (0.4–1.5)	.50
Married	Reference		Reference	
Separated	0.9 (0.1–9.2)	.96	1.0 (0.1–10.9)	.98
Divorced	1.5 (0.3–6.1)	.60	1.4 (0.3–5.8)	.62
Widowed	1.1 (0.5–2.5)	.77	1.1 (0.5–2.5)	.74
**Area of residence**				
Urban	Reference		Reference	
Rural	1.7 (1.3–2.3)	.001	1.6 (1.2–2.2)	.004
**Geographic region of residence**				
Red River Delta	20.7 (9.0–47.3)	<.001	29.4 (13.9–62.1)	<.001
Northern midland and mountain	16.2 (7.1–36.7)	<.001	22.8 (10.6–49.0)^f^	<.001
North central and central coastal	6.4 (2.8–14.6)	<.001	8.6 (4.0–18.4)^f^	<.001
Central highlands	3.3 (1.3–8.3)	.01	5.2 (2.2–12.3)^f^	<.001
South East	Reference		Reference	
Mekong River Delta^g^	0		0	
**Access to tobacco information**				
No	Reference		Reference	
Yes	1.1 (0.7–1.8)	.67	1.0 (0.6–1.7)	.96
**Access to health warnings**				
No	Reference		Reference	
Yes	0.8 (0.5–1.2)	.35	0.8 (0.5–1.2)	.27
**Access to tobacco advertisement**				
No	Reference		Reference	
Yes	0.9 (0.6–1.4)	.69	0.7 (0.4–1.1)	.09
**Believe smoking harms health**				
No	Reference		Reference	
Yes	2.1 (1.0–4.3)	.05	2.2 (1.0–4.8)	.06
**Believe smoking causes diseases**				
No	Reference		Reference	
Yes	0.2 (0.1–0.4)	<.001	0.3 (0.1–0.6)	.001
**Education^h^**				
Primary or less	NA		2.0 (1.0–4.1)	.06
Lower secondary			1.9 (0.9–3.8)	.08
Upper secondary			1.3 (0.7–2.6)	.41
Some college or more			Reference	

Abbreviations: OR, odds ratio; CI, confidence interval; NA, not applicable.

a χ^2^ test.

b Occupation based on reported main work status over the past 12 months of the respondent. Unstable jobs were defined as freelance worker and self-employed. This information is based on information in the asset wealth index.

c There were no students in this age group.

d This index was constructed by using household assets, utilities, and housing construction as variables in principal-components analysis and by computing a wealth index for each household surveyed.

e Based on the ethnic group that the respondent reported belonging to. Approximately 85% of the population of the country is Kinh.

f Significant at *P* < .05.

g No one in the sample population in the Mekong River Delta region reported waterpipe tobacco smoking.

h The highest educational level that the respondent completed: primary or less is equivalent to less than 6th grade; lower secondary is equivalent to finishing 6th to 9th grade; upper secondary is equivalent to finishing 10th to 12th grade; some college and above is study after upper secondary graduation.

## Discussion

Although the 6.4% prevalence of waterpipe smoking among adults in GATS Vietnam 2010 was slightly higher than the corresponding figures for Pakistan (6%) (4) and Tunisia (5.2%) (18), it was lower than the waterpipe use prevalence in Australia (11% in Arabic-speaking adults), Syria (9%–12%), and Lebanon (15%) (4,6,18). In addition, GATS Vietnam showed that the prevalence of waterpipe tobacco use was lower in 2010 than in 2002 through 2003 (6.4% vs 9%) (11). This decrease in waterpipe use rates, unlike the increasing trend in other countries (4,5), may suggest that the tobacco use control program in Vietnam has been successful in controlling waterpipe smoking. However, findings were not comparable because of different methods (ie, definition of smokers, geographic coverage, and sample size) (11). In 2001–2002, the Vietnam National Health Survey (VNHS) identified current waterpipe smokers as those who smoke from a waterpipe once a week (11). Therefore, GATS 2010 helps provide VINACOSH and the Ministry of Health with a baseline of waterpipe use for implementation of guidelines in the Framework Convention for Tobacco Control (16).

Waterpipe smokers have the perception that waterpipe tobacco has lower health risks and lower smoke toxicant levels than cigarette tobacco (7–9,19). However, our findings showed that those who believed that smoking causes diseases were less likely to engage in waterpipe smoking. The prevalence of waterpipe smoking among those who believed that smoking causes disease was lower than that among those who did not. This finding supports the recommendation for designing and implementing health education programs to increase the awareness of the adverse health effects of tobacco smoking in general and waterpipe smoking in particular in an effort to reduce waterpipe smoking in Vietnam.

Our study supports findings from other studies which report that more rural dwellers smoked waterpipe than those in urban areas (14,20). This finding may be associated with the findings that the prevalence of waterpipe smoking was higher among people with lower incomes than among those with the highest income (data not shown). We tested the interaction between residence and income (data not presented) and found that the prevalence of waterpipe smoking among poor people living in rural areas was twice the prevalence of the nonpoor people living in rural areas. This suggests that waterpipe tobacco control efforts should target rural areas and poor people in Vietnam. Furthermore, we found that waterpipe use was associated with ethnicity within 1 region of residence, particularly for the Dao and San Diu groups in the Northern midland and mountain region and the North central and central coastal area. This implies a cultural factor associated with waterpipe use in Vietnam that needs to be further explored in future studies.

Sex difference in the prevalence of waterpipe smoking found in this study was similar to findings from previous studies (6,9,10,18,21–26). A study in New Zealand (24) and another study in Turkey (6) found that sex, cigarette smoking, and the presence of waterpipe tobacco smokers among family members and friends have significant effects on the prevalence of waterpipe tobacco smoking. In the New Zealand study, area of residence, economic status of the family, and who the students lived with had no significant effect on the prevalence (24).

High prevalence of waterpipe tobacco smoking among student groups has been documented in previous studies (4,6,8,9,11,16,27). However, GATS Vietnam in 2010 found that students had the lowest waterpipe smoking prevalence. This finding may be associated with our finding that the prevalence of waterpipe tobacco smoking was lowest among those with some college education or more.

Our study has several limitations. First, data from the GATS on waterpipe tobacco smoking were self-reported; therefore, the practice may have been underreported. Second, GATS could not explore cultural factors associated with waterpipe use. Finally, the cross-sectional design of GATS does not allow us to establish any causal connection.

Our study presented updated data on prevalence of waterpipe tobacco smoking in Vietnam showing lower rates in 2010 than in 2001–2002. Area of residence, age group, asset-based wealth quintiles, geographic region of residence, and knowledge about the health effects of tobacco were significantly associated with waterpipe tobacco smoking status. Waterpipe use was most prevalent among undereducated, middle-aged male Vietnamese adults with low socioeconomic status. To reduce the spread of waterpipe tobacco use in Vietnam, waterpipe tobacco should be part of the tobacco use control program and should target the rural poor. Further studies are needed to examine perceptions of the adverse health effects of waterpipe use and the cultural characteristics of waterpipe smokers.
